# The NALP3 inflammasome is involved in neurotoxic prion peptide-induced microglial activation

**DOI:** 10.1186/1742-2094-9-73

**Published:** 2012-07-11

**Authors:** Fushan Shi, Lifeng Yang, Mohammed Kouadir, Yang Yang, Jihong Wang, Xiangmei Zhou, Xiaomin Yin, Deming Zhao

**Affiliations:** 1 State Key Laboratories for Agrobiotechnology, Key Lab of Animal Epidemiology and Zoonosis, Ministry of Agriculture, National Animal Transmissible Spongiform Encephalopathy Laboratory, College of Veterinary Medicine, China Agricultural University, Beijing, 100193, China

**Keywords:** Prion diseases, PrP106-126, NALP3 Inflammasome, IL-1β, Microglia

## Abstract

****Background**:**

Prion diseases are neurodegenerative disorders characterized by the accumulation of an abnormal disease-associated prion protein, PrP^Sc^. In prion-infected brains, activated microglia are often present in the vicinity of PrP^Sc^ aggregates, and microglial activation is thought to play a key role in the pathogenesis of prion diseases. Although interleukin (IL)-1β release by prion-induced microglia has been widely reported, the mechanism by which primed microglia become activated and secrete IL-1β in prion diseases has not yet been elucidated. In this study, we investigated the role of the NACHT, LRR and PYD domains-containing protein (NALP)3 inflammasome in IL-1β release from lipopolysaccharide (LPS)-primed microglia after exposure to a synthetic neurotoxic prion fragment (PrP106-126).

****Methods**:**

The inflammasome components NALP3 and apoptosis-associated speck-like protein (ASC) were knocked down by gene silencing. IL-1β production was assessed using ELISA. The mRNA expression of NALP3, ASC, and pro-inflammatory factors was measured by quantitative PCR. Western blot analysis was used to detect the protein level of NALP3, ASC, caspase-1 and nuclear factor-κB.

****Results**:**

We found that that PrP106-126-induced IL-1β release depends on NALP3 inflammasome activation, that inflammasome activation is required for the synthesis of pro-inflammatory and chemotactic factors by PrP106-126-activated microglia, that inhibition of NF-κB activation abrogated PrP106-126-induced NALP3 upregulation, and that potassium efflux and production of reactive oxygen species were implicated in PrP106-126-induced NALP3 inflammasome activation in microglia.

****Conclusions**:**

We conclude that the NALP3 inflammasome is involved in neurotoxic prion peptide-induced microglial activation. To our knowledge, this is the first time that strong evidence for the involvement of NALP3 inflammasome in prion-associated inflammation has been found.

## **Background**

Prion diseases, also known as transmissible spongiform encephalopathies, are fatal neurodegenerative disorders, characterized by brain vacuolation, neuronal cell death, and microgliosis [1]. They are caused by the conversion of cellular prion protein (PrP^C^) into the pathological isoform (PrP^Sc^) through conformational changes. PrP^Sc^ is protease-resistant, and has a higher proportion of β-sheet structure in place of the normal α-helix structure [2]. The accumulation of abnormal forms of prion protein (PrP^Sc^) has been shown to be the main causative agent of these diseases [3].

The neurotoxic PrP fragment 106–126 (PrP106-126) possesses similar physicochemical and pathogenic properties to PrP^Sc^, in that it forms amyloid fibrils with a high β-sheet content, shows partial proteinase K resistance, and is neurotoxic *in vitro*. Therefore, PrP106-126 is commonly used as a model for the investigation of PrP^Sc^ neurotoxicity [4-6].

A large number of studies have shown that the accumulation of aggregated PrP^Sc^ leads to activation of microglia, and these in turn produce chemotatic factors, pro-inflammatory cytokines, and neurotoxic factors [7-9]. Furthermore, studies on brains from prion-infected mice have found upregulation of multiple cytokines and chemokines, including interleukin (IL) 1β, tumor necrosis factor (TNF), and chemokine (C-C motif) ligand (CCL)3 [10].

IL-1β plays a central role in the regulation of immune and inflammatory responses. It is produced as the inactive precursor pro-IL-1β in the cytosol, and a variety of stimuli can lead to higher expression of pro-IL-1β [11]. The inactive pro-IL-1β can be cleaved by protease caspase-1 into the mature, active form, IL-1β. Several studies have shown that synthetic neurotoxic prion fragments activate mouse microglia and lead to an increase in the production of IL-1β *in vitro*[12-15]; however, the mechanism by which PrP106-126 induces IL-1β release is yet unknown.

The inflammasome is a cytosolic multiprotein complex that serves as a platform for activating the pro-inflammatory cytokines IL-1β and IL-18 via caspase-1 cleavage [16]. The inflammasomes play important roles in innate immunity pathways and are active players in inflammatory disorders. To date, several inflammasome complexes have been identified, of which the NACHT, LRR and PYD domains-containing protein (NALP) 3 inflammasome, (also known as NOD-like receptor family, pryin domain-containin (NLRP)3 or cold-induced autoinflammatory syndrome (CIAS)1), is probably the best studied [17,18]. This complex consists of the Nod-like receptor (NLR) NALP3, the apoptosis-associated speck-like protein (ASC), and pro-caspase-1, and can be activated by pathogen-associated molecular patterns and by endogenous danger signals [19,20].

In the present study, we investigated the role of the NALP3 inflammasome in PrP106-126-induced IL-1β release, and found that the NALP3-ASC inflammasome plays a key role in caspase-1 and IL-1β activation in microglia in response to PrP106-126 stimulation.

## **Methods**

### **Ethics Statement**

All of the animal experiments were conducted in accordance with the guidelines of Beijing Municipality on the Review of Welfare and Ethics of Laboratory Animals approved by the Beijing Municipality Administration Office of Laboratory Animals (BAOLA).

### **Reagents**

Rabbit anti-mouse caspase-1, NALP3, and ASC antibody were acquired from BioVision (Palo Alto, CA, USA), Abcam (Cambridge, MA, USA) and Santa Cruz Biotechnology (Santa Cruz, CA, USA), respectively. Rabbit anti-mouse nuclear factor (NF)-κB p65, Anti-mouse β-actin, and Max antibody were from Beyotime Biotechnology (Wuhan, Hubei, China), Lipopolysaccharide (LPS; *E. coli* L2630) and N-acetyl-cysteine (NAC, A9165) were from Sigma-Aldrich (St. Louis, MO, USA), ELISA kits for mouse interleukin 1β and the Fast Protein Precipitation and Concentration Kit were purchased from Wuhan Boster Biotech (Wuhan, Hubei, China). Reagents and apparatus used in immunoblotting assays were obtained from Bio-Rad (Hercules, CA, USA); the goat anti-rabbit secondary antibody was from Beyotime Biotechnology.

### **Isolation and culture of microglia cells**

Experiments were conducted on murine primary microglia and BV2 microglial cells. The choice of this cell line is justified by the close similarities between BV-2 and primary microglia in mechanisms mediating microglial activation [21]. Primary microglial cell cultures were obtained from neonatal C57BL/6 mice (5 to 7 days old) as described previously [14]. Briefly, after sterilization, the brain was dissected, then the cerebral cortices were collected and digested with trypsin (0.25 %) for 15 minutes at 37°C. The digested tissue was repeatedly sucked into a pipette to obtain single cells. The cells were then passed through a 200 μm mesh and separated by centrifugation at 100 *g* for 5 minutes. The mixed glial cells were cultured for about 6 to 7 days, after which the cells were suspended by agitation for 12 hours on a rotary shaker (180 rpm) at 37°C and transferred to another flask. After incubation for 2 hours at 37°C, microglia had adhered to the flask. The purity of the microglial cells was approximately 90 %, as determined using anti-CD-11b antibodies. BV2 cells, a murine microglial cell line, and ANA1, a murine macrophage cell line, were obtained (Xiehe Medical University, Yuqin Liu, Cell Culture Center, Beijing, China), and cultured in a humidified incubator at 37°C with 5 % CO2 in DMEM and F12 medium (Hyclone, Logan, UT, USA) supplemented with 10 % heat-inactivated FBS (Gibco, Grand Island, NY, USA), 100 μg/ml streptomycin, 100 U/ml penicillin (Gibco), and 2 mmol/l glutamine.

### **Prion protein peptide**

PrP peptides PrP106-126 and scrambled PrP106-126 (Scr-PrP) (sequences KTNMKHMAGAAAAGAVVGGLG and AVGMHAGKGLANTAKAGAMVG, respectively), were synthesized (Sangon Bio-Tech, China). The purity of prion peptides was >95 % according to the data from the synthesizer. The peptides were dissolved in 0.1 mol/l PBS to a concentration of 1 mmol/l, and left to aggregate at 37°C for 12 hours. Experiments were conducted with final peptide concentrations of 100 μmol/l.

### **Peptide treatment**

Microglia were primed with 300 ng/ml LPS for 3 hours, after which the culture medium was washed off and the cells were treated with the aggregated peptide PrP106-126 in culture medium. Scr-PrP was used as the negative control. Three wells were used for each group of experimental conditions. In experiments involving co-treatments with PrP106-126 plus high potassium (130 mmol/l) or NAC (15 mmol/l), cells were primed with 300 ng/ml LPS for 3 hours, and were then exposed to the indicated concentrations of the inhibitors.

### **Small interfering RNA transfections and treatments**

Small interfering (si)RNA used for NALP3 and ASC silencing, and the scramble siRNA sequence used as control (all Qiagen, Valencia, CA, USA) were used. For siRNA transfection, cells were plated at 0.8 × 10^5^ cells/well in a 12-well plate, and transfected the next day in accordance with the manufacturer’s instructions. Briefly, on the day of transfection, 75 ng siRNA (siRNA-NALP3, siRNA-ASC and control, respectively; Qiagen) were diluted in 100 μl culture medium without serum. A volume of 3 μl of transfection reagent (HiPerfect Transfection Reagent; Qiagen) was added to the diluted siRNA and mixed by vortex. The samples were then incubated for 5 to 10 minutes at room temperature to allow the formation of transfection complexes before adding the complexes onto the cells. The decrease in NALP3 and ASC expression after treatment was checked by quantitative PCR and western blot analysis.

### **Enzyme-linked immunosorbent assay for IL-1β secretion**

Cell-culture supernatants were assayed for IL-1β by ELISA using a commercial kit (Wuhan Boster Biotech) in accordance with the manufacturer’s instructions.

### **RNA isolation, complementary DNA synthesis and quantitative PCR**

Total RNA was extracted from cells using the SV Total RNA Isolation System (Promega, Madison, WI, USA), and reverse transcribed into complementary (c)DNA using a commercial kit (cDNA Synthesis Kit; Fermentas, Glen Burnie, MD, USA) using oligo (dT) 18 primers in accordance with the manufacturer’s instructions. Quantitative (q)PCR was performed using a commercial mix (SYBR Green Master Mix; Bio-Rad) and a thermal cycler (DNA Engine Opticon™ 2 system; MJ Research, Waltham, MA, USA) with the primers shown in Table 1. The amplification efficiency of these primers had been established by means of calibration curves. The total volume for qPCR was 20 μl, comprised of includes 8 μl water, 0.5 μl of each primer (10 μmol/l), 10 μl Master Mix and 30 ng of cDNA. The PCR amplification was as follows: after denaturation at 94°C for 2 minutes, 40 PCR cycles of 94°C for 20 seconds, 55°C for 20 seconds, 72°C for 20 seconds, followed by 1 cycle at 84°C for 1 second appended for a single fluorescence measurement above the melting temperature of possible primer-dimers. Finally, a melting step was performed consisting of 10 seconds at 70°C and slow heating at a rate of 0.1°C per second up to 95°C with continuous fluorescence measurement. Quantification was performed using the comparative C_T_ method (2^-ΔΔCT^) [22]. All samples were analyzed in triplicate.

**Table 1:** Primers used for quantitative PCR

**Name**	**Sequence (5′→3′)**
NALP3	ATTACCCGCCCGAGAAAGG
	TCGCAGCAAAGATCCACACAG
ASC	GCAACTGCGAGAAGGCTAT
	CTGGTCCACAAAGTGTCCTG
β-actin	GCTTCTTTGCAGCTCCTTCG
	CCTTCTGACCCATTCCCACC
CCL3	TCCCAGCCAGGTGTCATTT
	GGCATTCAGTTCCAGGTCAG
TNF	CCCTTCCTCCGATGGCTAC
	CGCCTCCTTCTTGTTCTGG

### **Extraction of nuclear and cytoplasmic protein and western blotting**

After treatment of microglia cells, the culture medium was discarded, and the cytoplasmic and nuclei proteins were extracted using a protein extract kit (Cytoplasmic and Nuclear Protein Extraction Kit; Wuhan Boster Biotech). Equal amounts of protein (40 μg in each lane) were separated by SDS-PAGE on 12 % gels, and the separated proteins were transferred onto a nitrocellulose membrane. Nonspecific binding sites were blocked by incubating the membrane with 5 % fat-free dried milk in Tris-buffered saline (TBS-T: 10 mmol/l Tris, 0.15 mol/l NaCL, 0.05 % Tween-20, pH of the solution adjusted to 7.5). Rabbit anti-NF-κB p65 (1:500), anti-caspase-1 (1:500), anti-NALP3 (1:5000), or anti-ASC (1:500) antibodies were added and incubated at 4°C overnight. Membranes were washed with TBS-T, and then incubated with the secondary antibody, either goat anti-mouse IgG or anti-rabbit IgG horseradish peroxidase-conjugated antibody (1:5000). Bands of immunoreactive protein were visualized after membrane incubation with enhanced chemifluorescence (ECF) reagent for 5 minutes, on an image system (Versadoc; Bio-Rad). The blot was stripped and reprobed with anti-β-actin (for cytoplasmic extracts) or anti-Max (for nuclear extracts) to estimate the total amount of protein loaded in gel.

### **Statistical analysis**

All assays were performed on three separate occasions. Data are expressed as means ± S.D. All comparisons for parametric data were made using Student’s *t* test or one-way ANOVA followed by post hoc Turkey’s test, Nonparametric data (ELISA of the primary microglia) were analyzed by the Kruskal–Wallis ANOVA test followed by the Nemenyi test for *post hoc* analyses. SPSS software (version 13.0: SPSS Inc., Chicago, IL, USA) was used, and *P* < 0.05 was considered significant.

## **Results**

### **Neurotoxic prion peptide PrP106-126 activates caspase-1 and induces interleukin-1β release in lipopolysaccharide-primed microglia**

**Figure 1:**
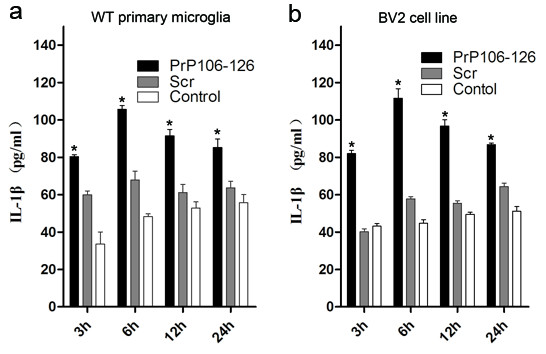
**PrP106-126 induces the release of interleukin (IL)-1β.** Cells were primed with 300 ng/ml lipopolysaccharide (LPS) for 3 hours, and then left untreated or stimulated with PrP106-126 (100 μmol/l) or Scr (100 μmol/l). Data show ELISA analysis of the release of IL-1β by **(a)** mouse primary microglia and **(b)** BV2 cell lines. Data represent the mean ± SD of triplicate samples from one of three independent experiments. *P < 0.05, significantly different from control and Scr-treated cells under the same experimental conditions.

To investigate the mechanisms of PrP106-126 induced release of IL-1β, we incubated primary mouse microglial cells and BV2 cell lines with PrP106-126 and its scrambled form, Scr-PrP. Because pro-IL-1β is not constitutively expressed in microglia [23], the cells were primed with 300 ng/ml LPS for 3 hours to induce pro-IL-1β synthesis and to mimic the chronic activation of microglia in prion disease. Treatment of LPS-primed BV2 microglia and primary microglia with PrP106-126 led to a significant increase in IL-1β release. At all time points examined, the IL-1β level was significantly higher in cells treated with PrP106-126 than in those treated with Scr-PrP or PBS (Figure 1).

**Figure 2:**
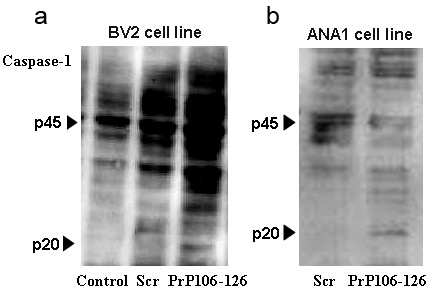
**PrP106-126 induces caspase-1 activation.** Cells were primed with 300 ng/ml lipopolysaccharide (LPS) for 3 hours and then left untreated or stimulated with PrP106-126 (100 μmol/l) or Scr (100 μmol/l). Data show western blot analysis of caspase-1 (p20) cleavage in **(a)** LPS-primed BV2 and **(b)** the ANA1 cell line. p45, full-length pro-form of caspase-1; p20, active subunit of caspase-1. Data represent one of three independent experiments.

Similarly, only the PrP106-126 treatment led to caspase-1 activation in LPS-primed BV2 microglia and the murine macrophage cell line, ANA1, as indicated by the cleavage of caspase-1 to its active p20 subunit. No caspase-1 cleavage was seen in LPS-primed BV2 microglia or in ANA1 treated with PBS or Scr-PrP (Figure 2). These results indicate that PrP106-126 induces IL-1β release and activates caspase-1 from microglia.

### **PrP106-126 upregulates NALP3 and ASC expression**

**Figure 3:**
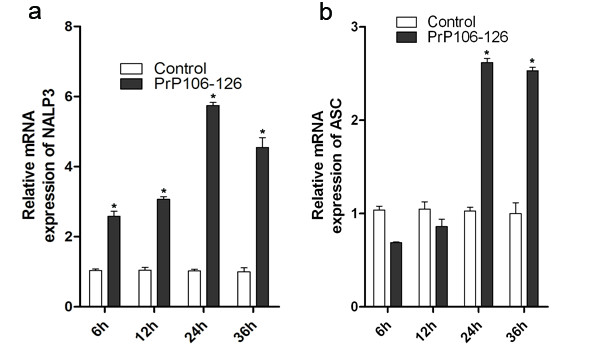
**Exposure of lipopolysaccharide-primed BV2 microglia to PrP106-126 increases mRNA expression of NACHT, LRR and PYD domains-containing protein (NALP)3 and ASC.** Measurement by quantitative PCR of the mRNA expression of **(a)** NALP3 and **(b)** ASC in BV2 microglia at the indicated time points after exposure to PrP106-126. Data were performed in triplicate and expressed as the mean ± SD, and are representative of three separate experiments. *P < 0.05, significantly different from control under the same experimental conditions.

To investigate the involvement of NALP3 inflammasome activation in PrP106-126-induced microglial activation, we first examined the effect of PrP106-126 treatment on the mRNA expression of NALP3 and ASC in BV2 microglia. PrP106-126 treatment significantly upregulated the mRNA expression of both NALP3 and ASC in microglia (Figure 3a,b). The mRNA level of NALP3 was significantly higher in PrP106-126-treated microglia than in PBS-treated microglia at all time points examined, while ASC expression was upregulated only at 24 and 36 hours after PrP106-126 stimulation. The PrP106-126-induced upregulation of NALP3 and ASC indicates an active participation of NALP3 inflammasome in PrP106-126-induced microglial activation.

### **PrP106-126-induced release of interleukin (IL)-1β requires the NALP3 inflammasome**

**Figure 4:**
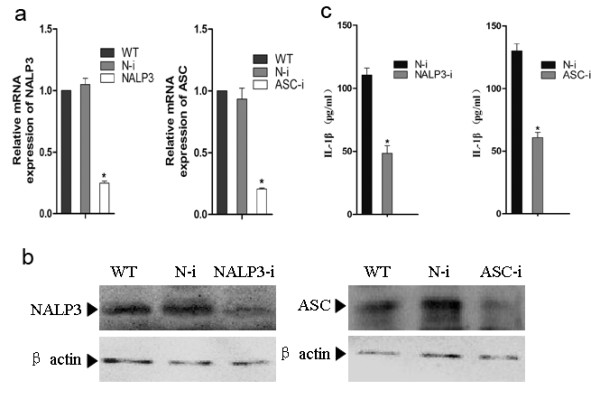
**PrP106-126-stimulated release of interleukin (IL)-1β is dependent on the NACHT, LRR and PYD domains-containing protein (NALP)3 inflammasome.** BV2 microglia cells were transfected with either control non-targeting small interfering (si) RNA (N-i), NALP3-targeting siRNA (NALP3-i), or ASC-targeting siRNA (ASC-i). **(a)** Quantitative PCR analysis of the mRNA expression of NALP3 and ASC in BV2 microglia after siRNA transfection. **(b)** Western blot analysis of the protein expression of NALP3 and ASC in BV2 microglia after siRNA transfection. **(c)** ELISA analysis of IL-1β released in the supernatants of BV2 microglia with NALP3 or ASC knock-down treated with PrP106-126. Data represent the mean ± SD of triplicate samples from one of three independent experiments. *P < 0.05, significantly different from control under the same experimental conditions.

To elucidate the role of the NALP3 inflammasome in PrP106-126-induced microglial activation, we examined the role of the NALP3 inflammasome in PrP106-126-induced IL-1β release. Because the NALP3 inflammasome is a multiprotein complex that consists of NALP3, ASC, and pro-caspase-1 [24,25], we analyzed the effect of siRNA-mediated disruption of NALP3 or ASC on IL-1β release in PrP106-126-treated microglia. The efficiency of siRNA-mediated disruption was evaluated at 24 and 48 hours after siRNA transfection by qPCR ( #x2009;4a) and western blot analysis (Figure 4b), respectively. Expression of NALP3 and ASC was significantly downregulated both at the mRNA (76 % and 80 %, respectively) and protein levels after siRNA transfection.

Following NALP3 or ASC disruption, BV2 cells were primed with 300 ng/ml LPS for 3 hours before PrP106-126 treatment. The cells were then exposed to PrP106-126, and the cell-culture supernatants were collected at 6 hours after PrP106-126 exposure and assayed for IL-1β by ELISA. Knock-down of either NALP3 or ASC significantly reduced the release of IL-1β after exposure to PrP106-126 (Figure 4c), suggesting a key role for the NALP3 inflammasome in PrP106-126- induced IL-1β release.

### **Increased levels of extracellular K + and N-acetyl-cysteine abrogate PrP106-126-induced secretion of interleukin-1β**

Several studies have shown that the NALP3 inflammasome assembly requires a low K + intracellular environment [26], and the activation of NALP3 inflammasome is reportedly blocked by reactive oxygen species (ROS) inhibitors through a mechanism that is not well understood [27]. To determine the role of K + and ROS in PrP106-126-induced NALP3 inflammasome activation, we evaluated the effect of increasing the level of extracellular K + and of NAC (an antioxidant known for its ability to scavenge ROS), on PrP106-126-induced secretion of IL-1β and NALP3 and ASC upregulation.

**Figure 5:**
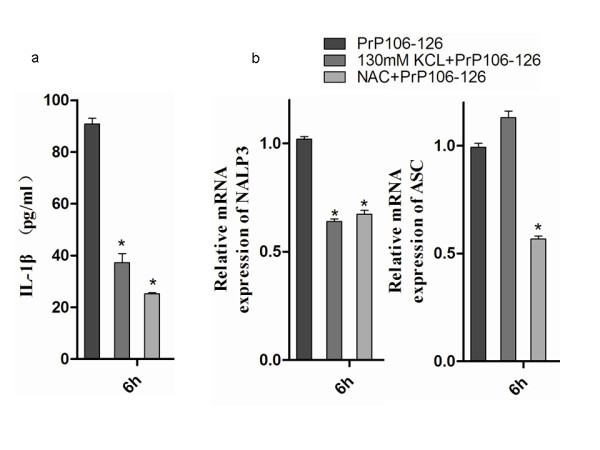
**High concentration of K + and N-acetyl-cysteine (NAC) blocks PrP106-126-stimulated release of interleukin (IL)-1β in BV2 microglia.** Lipopolysaccharide (LPS)-primed BV2 microglia were treated with PrP106-126 in combination with either K + or NAC for 6 hours, then the supernatants were collected for **(a)** ELISA analysis of the secreted IL-1β, and **(b)** qPCR analysis of the mRNA expression of NALP3 and ASC). Data were performed in triplicate and expressed as the mean ± SD, and are representative of three separate experiments. *P < 0.05, significantly different from PrP106-126 treated cells.

A high extracellular K + concentration significantly abrogated PrP106-126-induced release of IL-1β in LPS-primed microglia. Similarly, NAC significantly blocked IL-1β activation in LPS-primed microglia stimulated with PrP106-126 (Figure 5a).

NAC significantly abrogated PrP106-126-induced NALP3 and ASC upregulation; the mRNA levels of NALP3 and ASC significantly decreased after NAC treatment in PrP106-126-treated microglia and dropped to 68 % and 54 %, respectively, of the levels seen in microglia treated with PrP106-126 only (Figure 5b). Interestingly, higher levels of extracellular K + significantly reduced the PrP106-126-induced NALP3 upregulation (64 % of the mRNA level seen in microglia treated with PrP106-126 only) but had no effect on ASC upregulation (Figure 5b). These results suggest that a low K + intracellular environment and ROS production are also required for PrP106-126-induced NALP3 inflammasome activation, and indicate that the expression of ASC and NALP3 may be regulated through closely related but not identical regulatory pathways in PrP106-126-treated microglia.

### **The NALP3 inflammasome activation promotes tumor necrosis factor and chemokine (C-C motif) ligand 3 expression**

Previous studies with brain samples from mice infected with prion agent showed upregulation of multiple cytokines and chemokines, including pro-inflammatory TNF, IL-1α, transforming growth factor β, CCL2, and CCL3 [9]. To investigate the involvement of NALP3 inflammasome activation in PrP106-126-induced upregulation of pro-inflammatory cytokines and chemotactic factors, we examined the effect of siRNA-mediated silencing of NALP3 and ASC on the mRNA expression of TNF and CCL3 in LPS-primed BV2 microglia stimulated with PrP106-126.

**Figure 6:**
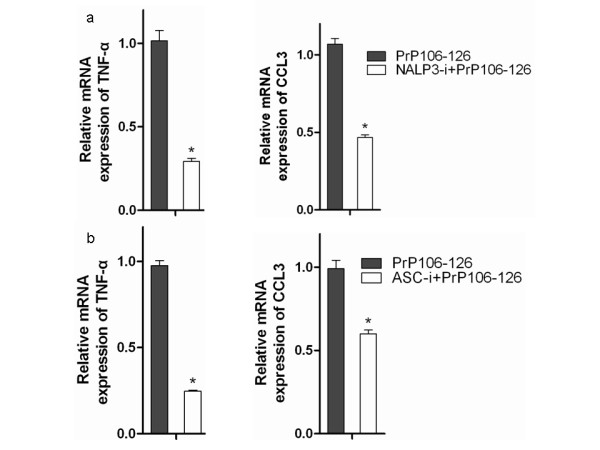
**The NALP3 inflammasome participates in tumor necrosis factor and chemokine (C-C motif) ligand (CCL)3 mRNA upregualtion in PrP106-126-stimulated microglia.** Real-time quantitative PCR analysis of the mRNA level of TNF-α and CCL3 in BV2 cells treated for 12 hours with PrP106-126 after small interfering (si)RNA-mediated silencing of **(a)** NALP3 and **(b)** ASC siRNA silencing. Data represent the mean ± SD of triplicate samples from one of three independent experiments. *P < 0.05, significantly different from PrP106-126 treated cells.

After siRNA-mediated disruption of NALP3 or ASC, cells were treated with PrP106-126 for 12 hours, then total RNA was extracted and used to measure the level of TNF and CCL3 encoding mRNA, using qPCR. Knock-down of either NALP3 or ASC significantly reduced PrP106-126-induced upregulation of TNF- and CCL3-encoding mRNA (Figure 6a,b). The degree of downregulation caused by NALP3 inflammasome disruption was more pronounced for TNF than for CCL3 expression. These data suggest that NALP3 inflammasome activation affects TNF and CCL3 expression in PrP106-126-stimulated BV2 cell line.

### **Nuclear factor-κB inhibition abrogates PrP106-126-induced upregulation of NALP3 and ASC expression**

**Figure 7:**
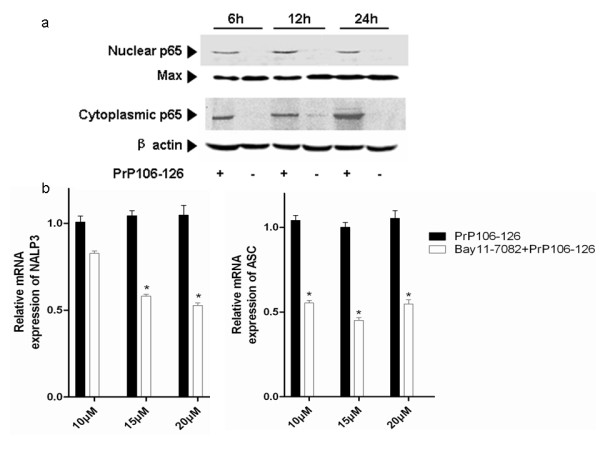
**Nuclear factor (NF)-κB activation is required for NACHT, LRR and PYD domains-containing protein (NALP)3-ASC inflammasome activation in PrP106-126-stimulated microglia.(a)** BV2 cells were stimulated with PrP106-126 (100 μmol/l) for 6, 12 and 24 hours, then the cytoplasmic protein and nuclear protein were collected and analyzed for NF-κB p65 translocation by western blot analysis. **(b)** BV2 cells stimulated with PrP106-126 were pretreated with Bay11-7082, a specific inhibitor of NF-κB (10, 15, and 20 μmol/l) or PBS (as a negative control) for 1 hour, and the mRNA expression of NALP3 and ASC was determined by quantitative PCR. Data represent the mean ± SD of triplicate samples from one of three independent experiments. *P < 0.05, significantly different from PrP106-126 treated cells.

The transcription factor NF-κB has been shown to play an important role in prion-induced inflammation, and PrP106-126-induced NF-κB activation has been widely reported [13,28,29]. In this study we also found that PrP106-126 treatment stimulated NF-κB activation as indicated by the nuclear translocation of p65 in BV2-microglia (Figure 7a).

We then examined the relevance of NF-κB activation to NALP3 activation through the analysis of the effect of NF-κB inhibitor, Bay 11–7082, on the expression of NALP3- and ASC-encoding mRNA in PrP106-126-treated BV2 microglia. The results showed that low, moderate, and high concentrations of Bay 11–7082 significantly reduced the PrP106-126-induced upregulation of NALP3- and ASC-encoding mRNA (Figure 7b), suggesting that NF-κB activation is required for NALP3 inflammasome activation.

## **Discussion**

Previous studies have shown that pathological prion protein can activate microglia and induce the release of inflammatory cytokines and chemokines *in vivo* and in *vitro*[9,12]. Furthermore, the inflammatory cytokine IL-1β has been shown to be an important factor in prion disease-associated inflammation [10]. Although it is clear that PrP^Sc^ can induce IL-1β from microglia both *in vivo* and *in vitro*, the mechanism of prion-mediated processing and release of IL-1β was unclear.

IL-1β is expressed as a biologically inactive pro-form, pro-IL-1β, in the cytoplasm of cells. Pro-IL-1β is the substrate of the cysteine protease caspase-1, which mediates the cleavage of pro-IL-1β and release of the mature, biologically active cytokine form of IL-1β. Caspase-1 itself is present as an inactive proform in the cytoplasm, and is activated by proteolytic self-processing. The induction of IL-1β secretion requires enhanced pro-IL-1β synthesis through transcriptional mechanisms via NF-κB, followed by a second stimulus that leads to the activation of caspase-1, processing of pro-IL-1β and release of mature IL-1β [17]. The NLR NALP3 interacts with the adapter protein ASC to form the inflammasome that has been identified as caspase-1 activator [17,30-33], and thus controls the second required step of IL-1β cytokine activation.

In the present work, we examined the signaling molecules that contribute to IL-1β activation in microglia in response to PrP106-126 stimulation. Our data indicate that the NLR protein NALP3 and the inflammasome adaptor ASC are involved in PrP106-126-induced caspase-1 and IL-1β activation. The siRNA-mediated disruption of either NALP3 or ASC significantly attenuated but did not completely abrogated IL-1β production, as residual IL-1β was still detected. This may be due to the incomplete silencing of NALP3 or ASC with siRNA-mediated disruption, or to the existence of alternative pathways for IL-1β activation. Indeed, recent reports have shown that inflammasome- and caspase-1-independent mechanisms may also be involved in the activation of IL-1β in microglia [34,35].

A recent study showed that Aβ, an endogenous peptide that forms insoluble fibrils in the brains of patients with Alzheimer’s disease (AD), activates the NALP3 inflammasome [36,37]. Activation of the NLRP3 inflammasome by islet amyloid polypeptide has been also reported in type 2 diabetes [36,37]. In the present study we found that the neurotoxic prion protein fragment PrP106-126, which forms amyloid fibrils with high β-sheet content, also activates the NALP3 inflammasome. This supports a key role for the NALP3 inflammasome as a general sensor for the recognition of peptide or protein aggregates that are involved in the pathogenesis of diseases such as AD, prion diseases, and systemic amyloidosis. However, it is not clear whether inflammasome activation has a beneficial or deleterious effect on the progression of amyloid-associated diseases.

Activation of NF-κB is known to be involved in PrP106-126 induced microglial activation [13,28,29]. Our experiments indicate that inhibition of NF-κB activation abrogates PrP106-126-induced NALP3 mRNA upregulation. This confirms that NF-κB activation acts upstream of NALP3, which is consistent with the well-described role of NF-κB as the main regulator of IL-1β precursor synthesis [38].

In the pathogenesis of prion diseases, several pro-inflammatory cytokines are upregulated, and are thought to play important roles in the recruitment and activation of microglia to areas in whcih amyloid aggregates are present. In this study, we found that the siRNA-mediated disruption of NALP3 and ASC significantly downregulated the mRNA expression of TNF-α and CCL3 in PrP106-126-stimulated microglia. This is consistent with previous reports on the role of inflammasome activation in promoting the production of proinflammatory factors [19,36]. It is likely that the upstream activation of NALP3 leads to the production of pro-inflammatory cytokines and chemotactic factors through the autocrine/paracrine effects of activated IL-1β.

It would be interesting to determine the relationship between NALP3 pathways and the other signaling pathways involved in PrP106-126-induced microglial activation. In a recent study, we showed that scavenger receptor CD36 participates in PrP106-126-induced microglial activation and that CD36 mediates PrP106-126-induced upregulation of IL-1β through a caspase-1-independent pathway suggesting that there is a limited interaction, if any, between inflammasome pathways and scavenger receptor-activated pathways during PrP106-126-induced microglial activation [35]. Further studies are required to elucidate the nature of the relationship between NALP3 activation and other signaling pathways involved in PrP106-126-induced microglial activation.

Among the models that have been put forth to explain the mechanisms of inflammasome activation is the efflux of K + and the possible influx of small danger-associated or pathogen-associated molecular patterns [24,26]. In the present study we found that hyperosmotic extracellular K + significantly attenuated PrP106-126-induced release of IL-1β through downregulation of NALP3 expression. The concentration of K + seems to be irrelevant for the regulation of ASC expression, as no significant change in the mRNA expression of ASC was seen in microglia treated with PrP106-126 in combination with K+. These results suggest that potassium efflux may account for inflammasome activation stimulated by neurotoxic prion peptides, and also indicate that the expression of ASC and NALP3 may be controlled through distinct regulatory pathways during the assembly and activation NALP3 inflammasome. This is consistent with the findings reported by Hanamsagar *et al*., who found that IL-1β processing in microglia is regulated by multiple pathways that differentially regulate ASC and NALP3 [34].

Another suggested mechanism for inflammasome activation is the generation of ROS. PrP106-126 is known to induce ROS production in treated microglia [27,30,39,40]. In the present study, we found that the ROS inhibitor NAC significantly reduced the production of IL-1β, and blocked NALP3 and ASC upregulation after exposure to PrP106-126, suggesting a role of ROS generation in the activation of the inflammasome in PrP106-126-stimulated microglia. Although it is not clear whether these mechanisms act in concert or independently, these results confirm the widely accepted view that no single mechanism can account for inflammasome activation [30].

Together our results demonstrate a role for NALP3 inflammasome in the mediation of IL-1β production after stimulation with neurotoxic prion peptides. Our results do not exclude the possible involvement of other inflammasome complexes in the activation of caspase-1 and IL-1β processing during the interaction between microglia and prion peptides. The inflammasomes harboring the NLR members NALP1, NALP3, IL-1-converting enzyme protease-activating factor (IPAF), and nucleotide-binding oligomerization domain-containing protein 2 are the best characterized, and, in certain pathological conditions, the assembly of inflammasomes harboring more than one NLR has been reported [41,42]. It would be therefore of interest to investigate the role of other inflammasome complexes, such as NALP1 and IPAF, in prion peptides-induced IL-1β production in microglia.

## **Conclusions**

We have identified a previously unrecognized role of NALP3 inflammasome as the main molecular platform responsible for IL-1β maturation and release in PrP106-126-stimulated microglia. Although more studies are needed *in vitro* and *vivo* to confirm and explore these initial findings, our study identified a potential molecular target for the modulation of prion-associated neuroinflammation through the modulation of the assembly of the NALP3 inflammasome.

## Abbreviations

AD = Alzheimer’s disease; ASC = Apoptosis-associated speck-like protein; CCL = Chemokine (C-C motif) ligand; cDNA = Complementary DNA; CIAS = Cold-induced autoinflammatory syndrome; DMEM = Dulbecco’s modified Eagle’s medium; ELISA = Enzyme-linked immunosorbent assay; FBS = Fetal bovine serum; IL = Interleukin; IPAF = Interleukin-1-converting enzyme protease-activating factor; LPS = Lipopolysaccharide; NAC = N-acetyl-cysteine; NALP = NACHT, LRR and PYD domains-containing protein; NF = Nuclear factor; NLRP = NOD-like receptor family, pryin domain-containing protein; PBS = Phosphate-buffered saline; qPCR = Quantitative polymerase chain reaction; ROS = Reactive oxygen specie; RT = Reverse transcriptase; SDS-PAGE = Sodium dodecyl sulfate polyacrylamide gel electrophoresis; siRNA = Small interfering RNA; TNF = Tumor necrosis factor.

## Misc

Fushan Shi and Lifeng Yang contributed equally to this work

## **Competing interests**

The authors declare that they have no competing interests.

## **Authors’ contributions**

FS designed the experiments with the help of LY, XZ, and MK, performed the experiments, analyzed the results, and drafted the manuscript. YY, JW, and XY performed the experiments of mouse primary microglia isolation. DZ secured funding for the project and helped with the final version of the manuscript. All authors read and approved the final manuscript.
